# Supplementary material for: Comparative multiomics analysis of cell physiological state after culture in a basket bioreactor
Source: Sci Rep. 2022 Nov 23;12:20161. doi: 10.1038/s41598-022-24687-4 (PMC9686226; doi:10.1038/s41598-022-24687-4)
Supplement: Supplementary file 1 — Supplementary Information 1. [file 41598_2022_24687_MOESM1_ESM.zip › raw data/Transcriptomic raw data/6.Enrichment/1.GO/TE1vsTC1/GSEA/TE1/ORGANIC_ANION_TRANSPORT/ORGANIC_ANION_TRANSPORT(GO_0015711).html]

Details for gene set ORGANIC\_ANION\_TRANSPORT(GO:0015711)[GSEA]


|  || Dataset | readcount.sample |
| Phenotype | sample.cls |
| Upregulated in class | TE1 |
| GeneSet | ORGANIC\_ANION\_TRANSPORT(GO:0015711) |
| Enrichment Score (ES) | 0.6467113 |
| Normalized Enrichment Score (NES) | 2.1973474 |
| Nominal p-value | 0.0 |
| FDR q-value | 0.47099996 |
| FWER p-Value | 0.0 |
Table: GSEA Results Summary

  

Fig 1: Enrichment plot: ORGANIC\_ANION\_TRANSPORT(GO:0015711)
  
Profile of the Running ES Score & Positions of GeneSet Members on the Rank Ordered List

  

| PROBE | DESCRIPTION   (from dataset) | GENE SYMBOL | GENE\_TITLE | RANK IN GENE LIST | RANK METRIC SCORE | RUNNING ES | CORE ENRICHMENT || 1 | 103215389 | SLC7A2 |  |  | 643 | 9.149 | 0.2661 | Yes |
| 2 | 103233435 | SLC7A5 |  |  | 1570 | 2.396 | 0.3020 | Yes |
| 3 | 103220154 | SLC1A4 |  |  | 1972 | 2.098 | 0.3517 | Yes |
| 4 | 103236270 | SLC7A11 |  |  | 2067 | 2.038 | 0.4132 | Yes |
| 5 | 103233242 | SLC7A6 |  |  | 2542 | 1.840 | 0.4513 | Yes |
| 6 | 103231465 | SLC7A8 |  |  | 2612 | 1.817 | 0.5068 | Yes |
| 7 | 103237078 | SLC7A13 |  |  | 2716 | 1.789 | 0.5599 | Yes |
| 8 | 103235521 | SLC1A5 |  |  | 2951 | 1.718 | 0.6048 | Yes |
| 9 | 103214212 | SLC7A1 |  |  | 3805 | 1.554 | 0.6168 | Yes |
| 10 | 103231442 | SLC7A7 |  |  | 4757 | 1.428 | 0.6203 | Yes |
| 11 | 103215141 | SLC1A3 |  |  | 5165 | 1.383 | 0.6467 | Yes |
| 12 | 103219505 | SLC1A1 |  |  | 11452 | 0.929 | 0.3958 | No |
| 13 | 103230647 | MPC2 |  |  | 13130 | 0.825 | 0.3475 | No |
| 14 | 103240904 | MPC1 |  |  | 13432 | 0.805 | 0.3600 | No |
| 15 | 103217283 | LOC103217283 |  |  | 17189 | 0.559 | 0.2102 | No |
| 16 | 103234451 | SLC7A9 |  |  | 18010 | 0.489 | 0.1893 | No |
| 17 | 103237679 | SLC1A2 |  |  | 20406 | 0.210 | 0.0891 | No |
| 18 | 103234456 | LOC103234456 |  |  | 22378 | 0.000 | 0.0011 | No |
Table: GSEA details
[plain text format]

  

Fig 2: ORGANIC\_ANION\_TRANSPORT(GO:0015711)
  
Blue-Pink O' Gram in the Space of the Analyzed GeneSet

  

Fig 3: ORGANIC\_ANION\_TRANSPORT(GO:0015711): Random ES distribution
  
Gene set null distribution of ES for
 **ORGANIC\_ANION\_TRANSPORT(GO:0015711)**
